# Obesity as an Important Marker of the COVID-19 Pandemic

**DOI:** 10.7759/cureus.21403

**Published:** 2022-01-19

**Authors:** Irfan A Mir, Renu Soni, Shrey K Srivastav, Inimerla Bhavya, Waseem Q Dar, Malik D Farooq, Vrinda Chawla, Mir Nadeem

**Affiliations:** 1 Internal Medicine, School of Medical Sciences & Research, Greater Noida, IND; 2 Department of Pulmonology, Mayo Institute of Medical Sciences, Lucknow, IND; 3 Otolaryngology, Head and Neck Surgery, Ministry of Health Saudi Arabia, Al Ula, SAU; 4 Internal Medicine, Government Medical College Srinagar, Srinagar, IND; 5 Internal Medicine, King Khalid University Hospital, Abha, SAU

**Keywords:** bmi, multiple co-morbidities, marker, obesity, covid 19

## Abstract

Introduction: In December 2019, the emergence of the new coronavirus disease 2019 (COVID-19) began in Wuhan, China. Thereafter, the disease has been spreading rapidly across the world, with about 300 million registered cases worldwide, and the numbers are also exponentially increasing in India, with about 34 million registered cases by the end of 2021. Among the comorbidities, obesity may increase the risk of hospitalization due to COVID-19 infection as it is related to immune system dysfunction. Since the epidemiological picture of COVID-19 is changing very rapidly. Therefore, it is very important to discuss the pattern of clinical manifestation and association with comorbidities. Hence, we have conducted this observational study in one of the tertiary care centers in North India.

Methods and Materials: We conducted a hospital-based prospective observational study in dedicated COVID-19 wards and ICU of a tertiary care center in North India with a sample size of 400 positive patients (males: 260, females: 140). We divided the patients in this study into three different age groups (less than 40 years, 40-60 years, and more than 60 years). The patients with age ≤ 18 years and BMI 18.5 kg/m2 were excluded from the study.

Results: Out of these 400 patients, 55 (13.8%) developed severe COVID-19. There was a fewer number of patients who developed severe COVID-19 in the normal and over-weight group. Moreover, obese patients progressed to more severe cases (34.5%). This also shows that after adjusting for age, compared to the normal-weight group, those who were overweight had a 1.48-fold chance of developing severe COVID-19 (OR 1.48, P 0.0455), while those who were obese had a 1.73-fold chance of developing the disease (ORs 1.73, P 5 0.0652). Regarding gender distribution, the association appeared to be stronger in men than in women. After similar adjustment, the ORs for overweight and obese patients compared to normal-weight patients were 1.39 (p 0.5870) and 3.55 (p 0.0113) in females and 1.36 (0.5115) and 6.19 (0.0001) in males, respectively.

Conclusion: Our study shows that obese patients with a BMI of greater than or equal to 27.5 are at higher risk of developing COVID-19 severity, especially in the male population. Moreover, severity may be related to other comorbid conditions. However, in our study, patients with chronic obstructive pulmonary disease (COPD) and GI/liver diseases were less obese, and severity was relatively low. So, the conclusion is that obese male patients with comorbidities are more likely to develop severe COVID-19 infection.

## Introduction

In December 2019, the emergence of the new coronavirus disease 2019 (COVID-19) began in Wuhan, China. Subsequently, the disease has been spreading rapidly, with about 300 million registered cases worldwide, and the numbers are also exponentially increasing in India, with about 34 million registered cases by the end of 2021 [1]. Among the comorbidities, obesity may increase the risk of hospitalization due to COVID-19 infection as it is related to immune system dysfunction [2,3]. Obesity results in a disproportionate share of total body oxygen consumption while breathing, leading to a decrease in functional residual capacity and expiratory volume [4]. Subsequent ventilation-perfusion abnormality can decrease ventilatory reserve and lead to respiratory failure in obese patients, even after minor pulmonary challenges [5,6]. In addition, patients with obesity have a higher risk of developing pulmonary embolism and aspiration pneumonia [7]. Obese patients were more likely to be admitted to the ICU for acute respiratory distress syndrome and to be mechanically ventilated and stay in the hospital longer than normal-weight patients [6]. According to the Indian Council of Medical Research-India Diabetes (ICMR-INDIAB) study 2015, the prevalence rate of obesity and central obesity in India ranges from 11.8% to 31.3% and 16.9%-36.3%, respectively [8].

As the epidemiological picture of COVID-19 is changing very rapidly, it becomes very important to discuss the pattern of clinical manifestation and association with comorbidities. Hence, this observational study has been conducted in one of the tertiary care centers in North India.

## Materials and methods

We conducted a hospital-based prospective observational study in dedicated COVID-19 wards and ICU of a tertiary care center in North India with a sample size of 400 positive patients (males: 260, females: 140). We divided the patients in this study into three different age groups (less than 40 years, 40-60 years, and more than 60 years). The patients with age ≤ 18 years and BMI ≤ 18.5 kg/m2 were excluded from the study. The criteria for ICU admission were based on the clinical characteristics of the patients at the time of admission. A patient with/without mild symptoms and normal vital signs was kept under constant observation in the separate wards for males and females. The medical records of the patients were analyzed by the research team of the medical department. Irrespective of clinical signs and symptoms, patients above 18 years of age with laboratory-confirmed COVID-19 infection were included. Verbal informed consent was obtained from the patients. The COVID-19 diagnosis was confirmed by a positive high-throughput sequencing test or a real-time reverse transcription-polymerase chain reaction test (RT-PCR) for nasal and throat swabs. 

Data collection

Epidemiological, clinical, laboratory, radiological, treatment, and outcome characteristics were collected using data collection forms from electronic medical records and patient history. All data were reviewed by internal medicine specialists. Information collected included demographic data, medical history, and clinical examination, specifically BMI, exposure history, underlying comorbidities, symptoms, signs, laboratory, and radiographic findings. COVID-19 severity was determined as per the protocol of the Ministry of Health and Family Welfare, Government of India [9]. All patients were categorized into three BMI categories as per the guidelines of WHO: normal (18.5-23 kg/m2), overweight (23-27.5 kg/m2), and obese (≥27.5 kg/m2) [10]. Each participant gave written informed consent to participate in the study. The study protocol was approved by the institutional review board and institutional ethics committee.

Statistical analysis 

Data analysis was performed using IBM Corp. Released 2011. IBM SPSS Statistics for Windows, Version 20.0. Armonk, NY: IBM Corp. Continuous data were summarized as mean and standard deviation. Results on categorical variables were described as frequency and percentage, and their comparison was performed using the chi-square test or Fisher's exact test when cell counts were small. Univariable logistic regression was used to examine the age-adjusted model with disease severity, using odds ratios (ORs). A p-value of < 0.05 was considered statistically significant. 

## Results

As seen in Table 1, patients who initially presented with cough, fever, headache, dyspnoea, vomiting, chest and abdominal pain, and altered mental status developed more severe COVID-19 illness than the patients who initially presented with a sore throat, myalgias, nasal congestion, loss of smell, and loss of taste (p-value ranges from < 0.0001 to 0.9).

**Table 1:** Severity of COVID-19 according to initial symptoms

Initial Symptoms		Non severe	Severe			p-valve	
Cough	Yes 200	155	45 (22.5%)			<0.0001	
	No 200	190	10 (5%)				
Fever	Yes 270	219	51 (18.9%)			<0.0001	
	No 130	126	4 (3%)				
Sore throat	Yes 280	248	32 (11.4%)			0.05	
	No 120	97	23 (19.1%)				
Myalgia	Yes 284	246	38 (13.4%)			0.7	
	No 116	99	17 (14.6%)				
Headache	Yes 270	223	47 (17.4%)			0.002	
	No 130	122	8 (6.1%)				
Nasal congestion	Yes 140	117	23 (16.4%)			0.42	
	No 260	128	32 (25%)				
Dyspnoea	Yes 54	27	27 (50%)			0.000	
	No 346	318	28 (8%)				
Vomiting	Yes 30	20	10 (33.3%)			0.001	
	No 370	325	45 (14%)				
Chest pain	Yes 15	11	4 (26.6%)			0.27	
	No 385	334	51 (13.2%)				
Abdominal pain	Yes 17	9	8 (47%)			<0.001	
	No 383	336	47 (12.2%)				
Loss of smell	Yes 53	46	7 (13.2%)			0.9	
	No 347	299	48 (14%)				
Loss of taste	Yes 77	69	8 (10%)			0.34	
	No 323	276	47 (14.5%)				
Altered mental status	Yes 3	0	3 (100%)			<0.001	
	No 397	345	52 (13%)				

**Figure 1:**
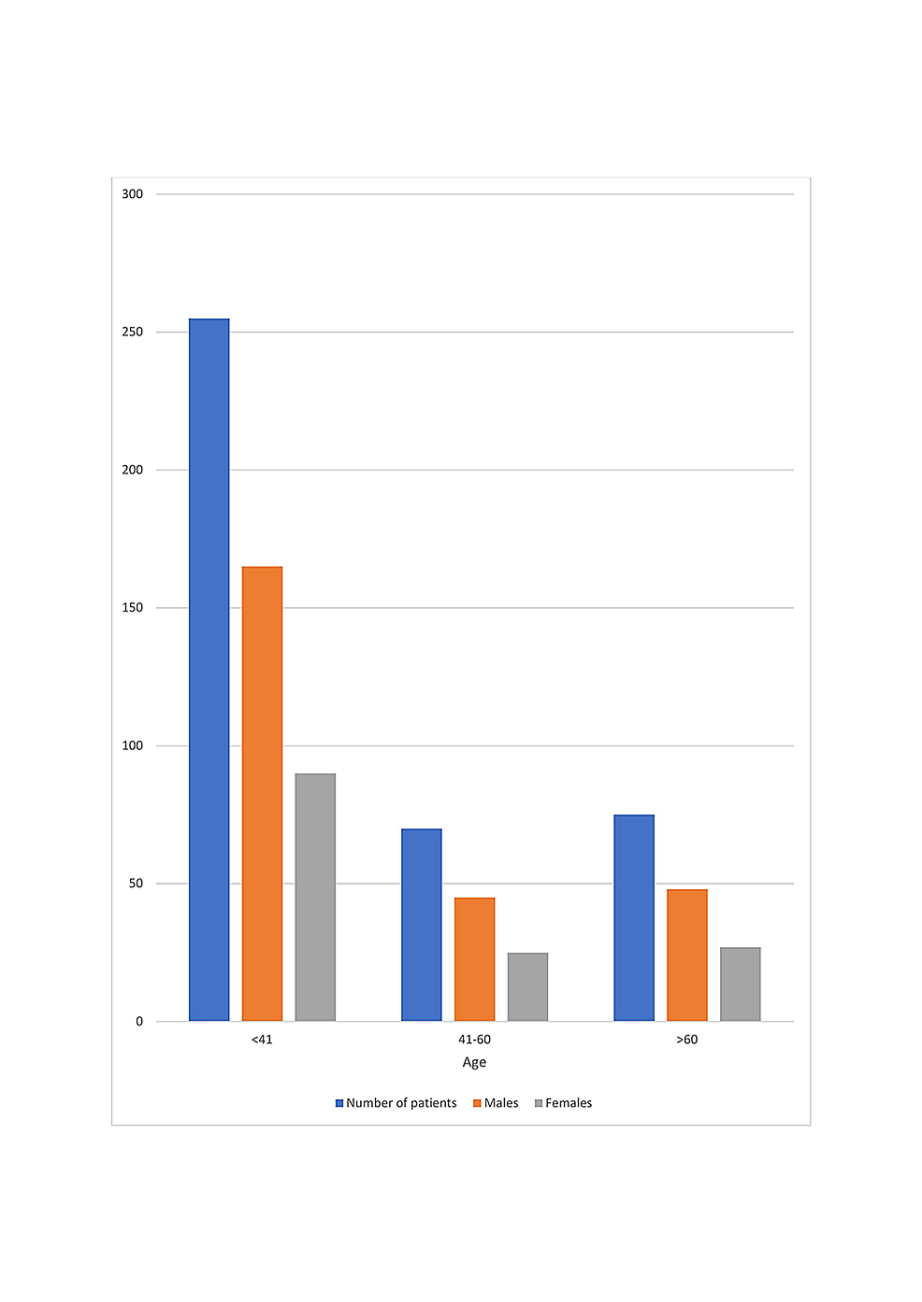
The distribution of patients according to age and sex

The distribution of these patients as per their age and sex can be seen in Figure 1.

Table 2 shows that patients with underlying diseases such as diabetes, hypertension, TB (tuberculosis)/Hx (history of TB), asthma, renal disease, HIV (Human immunodeficiency virus), and cancer tended to develop more severe COVID-19 (P-value of, < 0.001 to 0.01). However, patients with COPD (chronic obstructive pulmonary disease) (p-value 0.001) and gastrointestinal (GI)/liver (p-value 0.21) diseases were relatively less likely to develop severe COVID-19 disease.

**Table 2:** Severity of COVID-19 according to multiple co-morbidities

Co-morbidity		Non severe	Severe	p-value
Diabetes Mellitus	Yes 38	16	22 (57%)	<0.0001
	No 362	329	33 (9%)	
Hypertension	Yes 52	23	29 (55.7%)	<0.0001
	No 348	322	26 (7%)	
TB/Hx of TB	Yes 14	4	10( 71.4%)	<0.0001
	No 386	341	45 (11%)	
Asthma	Yes 5	1	4 (80%)	<0.001
	No 395	344	51 (13%)	
COPD	Yes 28	18	10 (35%)	0.001
	No 372	327	45 (12%)	
Renal Disease	Yes 20	8	12 (60%)	<0.0001
	No 380	337	43 (11.3%)	
HIV	Yes 2	0	2 (100%)	0.01
	No 398	345	53 (13.3%)	
Gastrointestinal tract (GI)/Liver disease	Yes 14	10	4 (28%)	0.21
	No 386	335	51 (13.2%)	
Carcinoma	Yes 5	1	4 (80%)	<0.001
	No 395	344	51 (13%)	

Of the 400 patients, 38.7% were of normal weight, 41% were overweight, and 20% were obese. The obese group had a higher percentage of diabetes, hypertension, kidney disease, gastrointestinal/liver disease (p-value of < 0.0001 to 0.5). The overweight group had a higher percentage of asthma (p-value 0.8), and the normal BMI group had a higher percentage of COPD (p-value 0.63) and tuberculosis (TB) /history of TB (p-value 0.5) (Table 3).

**Table 3:** Association of co-morbidities with BMI (Kg/m2)

		BMI (kg/m^2^)		Yates p-value
Comorbidities	Normal (18.5-23) (n=155)	Overweight (23-27.5) (n=164)	Obese ( ≥27.5) (n=81)	
Diabetes (n=38)	8/38 (21%)	12/38 (31.5%)	18/38 (47%)	0.001
Hypertension(n=52)	6/52 (11.5%)	19/52 (36.5%)	27/52 (52%)	<0.0001
Asthma (n=5)	1/5 (20%)	3 (60%)	1/5 (20%)	0.8
COPD (n=28)	12/28 (42.8%)	9/28 (32%)	7/28 (25%)	0.63
Renal Disease (n=20)	3/20 (15%)	8/20 (40%)	9/20 (45%)	0.03
GI/Liver Disease (n=14)	2/14 (14%)	4/14 (28%)	8/14 (57%)	0.01
Carcinoma (n=5)	1/5 (20%)	2/5 (40%)	2/5 (40%)	0.8
HIV (n=2)	½ (50%)	½ (50%)	0	0.8
TB/History of TB (n=14)	7/14 (50%)	3/14 (21%)	4 (28.5%)	0.5

As shown in Table 4, out of 400 patients, 55 (13.8%) progressed to severe COVID-19. Some fewer patients developed severe COVID-19 in the normal and over-weight group. Moreover, obese patients progressed to more severe cases (34.5%). This also shows that after adjusting for age, compared with the normal weight group, those who were overweight had 1.48-fold odds of developing severe COVID-19 (OR 1.48, P 0.0455), while those who were obese were at 1.73-fold odds of developing the disease (OR 1.73, P 5 0.0652).

**Table 4:** Association b/w BMI & COVID-19 severity

BMI, kg/m^2^	Number/Total, (%)	Age-adjusted model (ORs)	P-value
Total	11/155 (7.1)	1.00	
18-23			
23-27.5	16/164 (9.75)	1.48	0.0455
			³ 27.5	28/81 (34.57)	1.73	0.0652
Men	7/102 (6.86)	1.00	
18-23			
24-27.5	10/107 (9.35)	1.36	0.5115
³ 27.5	17/40 (42.5)	6.19	0.00001
Women	4/53 (7.55)	1.00	
18-23			
24-27.5	6/57 (10.53)	1.39	0.5870
³ 27.5	11/41 (26.83)	3.55	0.0113

Regarding the sex distribution, the association appeared to be more pronounced in men than in women. After similar adjustment, the ORs for overweight and obese patients versus normal-weight patients were 1.39 (p 0.5870) and 3.55 (p 0.0113) in women, respectively, and 1.36 (0.5115) and 6.19 (0.0001) in men, respectively (Table 4).

This section of the paper comprehensively illustrates all the relevant outcomes extracted from the research through the tabular form.

## Discussion

We conducted a hospital-based prospective observational study in dedicated COVID-19 wards and intensive care units of a tertiary care center. A total of 400 patients aged greater than 18 years were admitted to the hospital over three months. Of the 400 positive cases, 260 were male, and 140 were female. We found that the male gender predominated in COVID-19 infections. Similar results are shown by Marco Cascella et al.'s [11], Gebhard C et al.'s [12], and Jin JM et al.'s [13] studies. According to age distribution, 255 patients were between < 41 years, 70 were between 41-60 years, and 75 patients were > 60 years. Thus, more young patients were infected with COVID-19 (Figure 1). This is in agreement with S. Jakhmola et al.'s [14] study. During the COVID-19 pandemic, patients were hospitalized with various symptoms. In our study, the patients who presented with cough, fever, headache, vomiting, chest pain, abdominal pain, and altered mental status developed severe COVID-19 infection. However, patients with altered mental status, dyspnoea, and abdominal pain have a very high probability of developing COVID-19 infection (p- < 0.001) (Table 1). Similarly, Qingxian Cai et al.'s [15] and Naila Shoaib et al.'s [16] studies showed that fever and cough as initial symptoms were more likely to lead to severe COVID-19. 

Although the respiratory system is the main target of SARS-CoV-2, it can also affect other major organ systems such as the gastrointestinal tract (GI), hepatobiliary, cardiovascular, renal, and central nervous systems. Therefore, the severity of COVID-19 also depends on the other comorbid conditions. In our study, we found that patients with underlying diseases like diabetes, hypertension, renal disease, asthma, active or previous tuberculosis (both pulmonary and extrapulmonary), and carcinoma were more severely affected (Table 2). This is also evident from Qingxian Cai et al.'s [15] and Stokes EK et al.'s [17] studies.

Our study observed that obese patients had more comorbid diseases than over-weight and patients with normal BMI (Table 3), which is also consistent with Ciro Andolfi et al.'s [18] study. However, we observed that among the patients with COPD and GI/liver diseases, less than 30% were obese, and the severity of COVID-19 was relatively less than other comorbid conditions (Tables 2, 3). 

Table 4 concludes that obese patients tend to develop more severe COVID-19 disease than overweight and normal BMI patients. Among them, the male population is capable of developing higher severity. In addition to the above studies, obesity may increase the risk of severe COVID-19 disease, as excessive weight gain may increase the risk of community-acquired pneumonia [19]. 

The limitations of our study are the small patient size and short duration. In addition, we did not consider all other comorbidities and the sample size was too small for some comorbid patients.

## Conclusions

Our study shows that obese patients with a BMI of ≥ 27.5 are at higher risk of developing COVID-19 severity, especially in the male population. Moreover, severity may be related to other comorbid conditions. However, in our study, patients with COPD and GI/liver diseases were less obese, and severity was relatively low. Hence, it is concluded that obese male patients with comorbidities are more likely to develop severe COVID-19 infection.
